# Is tumour volume an independent predictor of outcome after radical prostatectomy for high-risk prostate cancer?

**DOI:** 10.1038/s41391-021-00468-4

**Published:** 2021-11-29

**Authors:** Nicholas Raison, Pol Servian, Amit Patel, Ainkaran Santhirasekaram, Andrew Smith, Maidie Yeung, Josephine Lloyd, Ethna Mannion, Andrea Rockall, Hashim Ahmed, Mathias Winkler

**Affiliations:** 1 Imperial Urology, Charing Cross Hospital, Imperial College Healthcare NHS Trust, London, UK; 2 MRC Center for Transplantation, King’s College London, London, UK; 3 Department of Urology, Hospital Germans Trias i Pujol, Autonomous University of Barcelona, Barcelona, Spain; 4 Imperial Prostate, Division of Surgery, Department of Surgery and Cancer, Faculty of Medicine, Imperial College London, London, UK; 5 Vattikuti Urology Institute, Henry Ford Hospital, Detroit, MI USA; 6 Department of Surgery and Cancer, Imperial College Healthcare NHS Trust, London, UK; 7 Department of Computing, Imperial College London, London, UK; 8 North West London Pathology, Charing Cross Hospital, Imperial College Healthcare NHS Trust, London, W2 1NY UK; 9 Division of Cancer, Department of Surgery and Cancer, Faculty of Medicine, Imperial College London, London, UK

**Keywords:** Translational research, Cancer therapy

## Abstract

**Background:**

Preoperative PSA, ISUP grade group (GG), prostate examination and multiparametric MRI (mpMRI) form the basis of prostate cancer staging. Unlike other solid organ tumours, tumour volume (TV) is not routinely used aside from crude estimates such as maximum cancer core length. The aim of this study is to assess the role of TV as a marker for oncological outcomes in high-risk non-metastatic prostate cancer.

**Methods:**

A prospectively maintained database of patients undergoing minimally invasive (laparoscopic or robot-assisted laparoscopic) radical prostatectomy at a UK centre between 2007 and 2019 were analysed. A total of 251 patients with NCCN high or very high-risk prostate cancer were identified. Primary outcome measure was time to biochemical recurrence (BCR) and the secondary outcome was time to treatment failure (TTF). TV was measured on the pathological specimen using the stacking method. Multivariable cox regression analysis was used to identify factors predicting BCR and TFF. TV as a predictor of BCR and TFF was further analysed through time-dependent receiver operating characteristic (ROC) curves. Kaplan–Meier survival estimates were used to evaluate TV cut-off scores.

**Results:**

Median follow up was 4.50 years. Four factors were associated with BCR and TFF on multivariable analysis (TV, pathological GG, pathological T stage, positive margin >3 mm). Area under the Curve (AUC) for TV as a predictor of BCR and TTF at 5 years was 0.71 and 0.75, respectively. Including all 4 variables in the model increased AUC to 0.84 and 0.85 for BCR and TFF. A 2.50 cm TV cut off demonstrated a significance difference in time to BCR, *p* < 0.001.

**Conclusions:**

Pathological tumour volume is an independent predictor of oncological outcomes in high risk prostate cancer but does not add significant prognostic value when combined with established variables. However, the option of accurate TV measurement on mpMRI raises the possibility of using TV as useful marker for preoperative risk stratification.

## Introduction

Staging and planning of radical prostatectomy is based on preoperative PSA, ISUP grade group, prostate examination and multiparametric MRI (mpMRI). Just as the use of mpMRI improved diagnostic accuracy, it refined prostate cancer staging. While this undoubtedly reduced staging errors and improved selection for nerve-sparing techniques its full potential for pre- and post-operative prognostication has not yet been harnessed. Important staging parameters such as extracapsular extension and seminal vesicle invasion form the basis of various prognostic classifications in addition to PSA, GG and biopsy information [1]. Tumour volume is a parameter that features in many pre- and post-operative staging classifications for solid organ cancers including breast, testis and kidney. In prostate cancer only crude pre-operative surrogates of pathological tumour volume like maximum cancer core length or percentage of positive biopsy cores have been used. Indeed, despite the utility of mpMRI, it is not recognised in staging schema. Hitherto, tumour volume as the simplest first degree radiomic feature was difficult to measure on staging mpMRI. This is now being revisited. Use of machine learning approaches in addition to radiomics analysis has led to the successful segmentation of the prostate offering the possibility of TV as one of the simplest first degree radiomic features for use as a prognostic parameter [2]. In this context we firstly explore the correlation of pathological tumour volume with oncological outcome [3].

In prostate cancer, Stamey et al. first demonstrated that TV was a possible predictor for capsular penetration, seminal vesicle invasion and metastatic disease [4]. Subsequent studies have focussed on using TV to identify insignificant prostate cancers with very few studies including high-risk tumours. Classically a cut off volume of 0.50 cm^3^ has been used to classify clinically insignificant cancer [5, 6].

If size reflects biological potential, we hypothesize that TV as measured on a pathological specimen adds important information on tumour biology and aids classification and planning of treatment [7]. This study aims to assess the role of TV as a marker for cancer recurrence in high-risk non-metastatic prostate cancer. It is thus considered as the initial experimental step towards using segmented TV combined with other radiomic features on mpMRI as a prognostic staging parameter.

### Subjects and methods

We analysed a prospectively maintained database of patients undergoing minimally invasive (laparoscopic or robot assisted laparoscopic) radical prostatectomy at a tertiary UK referral centre between 2007 and 2019. All patients with NCCN high-risk or very high-risk (forthwith termed high-risk) prostate cancer were identified. Only patients with complete information on ISUP grade group (GG), PSA, tumour stage, TV and outcome data were included. TV was calculated from the final pathological specimen using the stacking method which provides an accurate three-dimensional reconstruction of the tumour and cubic tumour volume [8]. For multifocal tumours, the total aggregate volume was reported. All patients underwent DRE, PSA, prostate MRI and prostate biopsy according to practice at the time (transrectal ultrasound guided, transperineal template ultrasound guided or MRI targeted transperineal ultrasound guided). Patients were excluded if they had evidence of pelvic lymph node or metastatic disease on preoperative evaluation. Patients undergoing salvage prostatectomy following radiotherapy or focal therapy were excluded.

Follow up included PSA at least every 6 months for 2 years and annually thereafter. The primary outcome measure was time to biochemical recurrence (BCR) from the date of radical prostatectomy. BCR was defined as a PSA rise above 0.20 ng/ml or physician defined (three consecutive PSA rises above nadir). The secondary outcome measure was Time to Treatment Failure (TTF) from the date of radical prostatectomy. Treatment failure was defined as the use of any form of salvage or palliative treatment, occurrence of local recurrence or distant metastasis as diagnosed on imaging. There were no deaths attributable to prostate cancer without preceding BCR and adjuvant or salvage treatment.

### Statistical analysis

Data were shown to have non-Gaussian distributions and non-parametric analyses were performed. Medians and interquartile ranges were reported for continuous variables.

Multivariable Cox regression analyses were performed to predict BCR and TFF free survival. A model was constructed a-priori containing TV in addition to the principles variables predicting oncological outcomes based on current literature and agreed by the study authors [9]. Included variables were pathological GG, pathogical T stage, presenting PSA, age, margin status (positive >3 mm), Charlson Comorbidity score and TV. Significance was set at *p* < 0.050.

The diagnostic capability of TV was analysed through time-dependent receiver operating characteristic (ROC) curves with a Kaplan–Meier estimate of time. Censored survival data was plotted using the survivalROC package for R (R Core Team 2020. R: A language and environment for statistical computing. R Foundation for Statistical Computing, Vienna, Austria) [10]. ROC curves were plotted for TV alone and for all variables in the final multivariable Cox’s proportional hazards regression model. To accommodate multiple covariates, a fitted linear predictor from the Cox model was used to construct the ROC curve [11].

Area under the Curve (AUC) was used to assess the overall diagnostic accuracy of TV. Youden’s index was used to identify the optimum cut-off. TV cut off values were compared used the Kaplan–Meier estimate with a log rank test for time to BCR and treatment failure. All other statistical analyses were performed using SPSS (IBM Corp. Released 2017. IBM SPSS Statistics for Windows, Version 25.0. Armonk, NY: IBM Corp).

## Results

A total of 685 patients underwent minimally invasive radical prostatectomy and met the inclusion criteria for the study: 251 patients had high-risk prostate cancer. Patient demographic, clinical and tumour characteristics for high-risk and non-high-risk populations are reported in Table 1. Median follow up in the high-risk cohort was 4.50 years and 45% (*n* = 112) of patients had over 5 years follow up. Aside from tumour features, clinical and demographic details for patients with and without high-risk prostate cancer were equivalent. TV were significantly greater in high-risk than non-high-risk patients despite equal overall specimen sizes. 14 patients died during follow up.

**Table 1:** Demographic and clinical characteristics of patients with high-risk and non high-risk prostate cancer.

Parameter	High risk prostate cancer	Non high-risk prostate cancer	*P* value
Total Patients (*n*)	251	434	
Median Age (IQR)	63.00 (8.20)	62.90 (8.60)	0.290
Median PSA (IQR)	11.90 (12.00)	8.00 (4.56)	<0.001
Mean Charlson Comorbidity Index (IQR)	0 (1)	0 (1)	0.812
Median Tumour Volume (IQR)	3.60 (5.45)	1.93 (2.55)	<0.0001
Median No. of Weeks follow-Up (IQR)	232.00 (310.00)	258.50 (275.70)	0.372
Median Specimen Size (IQR)	41.00 (21.00)	40.00 (19.00)	0.144
Patients with Neoadjuvant Treatment (ADT and/or Radiotherapy) (%)	63 (25.10%)	19 (4.38%)	<0.001
Pathological Grade Group, *n* (%)			
1	7 (2.79)	70 (16.13)	<0.001
2	104 (41.43)	280 (64.52)	
3	86 (34.26)	75 (17.28)	
4	12 (4.78)	4 (0.92)	
5	42 (16.73)	5 (1.15)	
Pathological T Stage, *n* (%)			
T2a	12 (4.78)	17 (3.933)	<0.001
T2b	3 (1.20)	8 (1.85)	
T2c	64 (25.50)	258 (59.58)	
T3a	113 (45.02)	114 (26.33)	
T3b	54 (21.51)	32 (7.39)	
T4	5 (1.99)	4 (0.92)	
Biochemical Recurrence, *n* (%)	137 (54.58)	147 (33.87)	<0.001
Failure Free Survival, *n* (%)	132 (52.59)	314 (72.35)	<0.001

Following multivariable analysis predicting BCR and TFF using a model containing seven predictors as described above, four variables remained significant for predicting both BCR and TFF (Table 2). These were TV, pGG, pT and positive margin >3.00 mm.

**Table 2:** Multivariable analyses of factors predicting BCR and TTF in patients with high-risk prostate cancer.

Covariate	Multivariable analysis for time to BCR		Multivariable analysis for time to TFF	
	Hazard Ratio (95% CI)	*p* value	Hazard Ratio (95% CI)	*p* value
Age	0.98 (0.95–1.02)	0.322	0.99 (0.95–1.02)	0.411
PSA	0.99 (0.97–1.00)	0.117	0.98 (0.96–1.00)	0.100
Tumour Volume	1.06 (1.03–1.10)	<0.001	1.07 (1.03–1.11)	<0.001
Pathological Grade Group		0.03	n/a	0.018
Grade Group 1	reference	n/a	reference	n/a
Grade Group 2	1.96 (0.47–8.28)	0.358	2.62 (0.35–19.47)	0.345
Grade Group 3	2.37 (0.56–10.11)	0.242	3.42 (0.46–25.61)	0.229
Grade Group 4	4.19 (0.83–21.31)	0.084	5.24 (0.57–47.80)	0.142
Grade Group 5	4.02 (0.93–17.48)	0.063	6.16 (0.81–46.71)	0.079
Pathological T Stage		0.01	n/a	0.01
T2a	reference	n/a	reference	n/a
T2b	2.43 (0.21–27.50)	0.475	3.45 (0.24–49.49)	0.362
T2c	1.77 (0.50–6.22)	0.374	1.90 (0.41–8.69)	0.412
T3a	4.17 (1.24–14.02)	0.021	5.39 (1.25–23.28)	0.024
T3b	5.33 (1.58–18.04)	0.007	6.92 (1.60–30.01)	0.010
T4	9.54 (1.83–49.70)	0.007	10.55 (1.69–65.85)	0.012
Positive Margin Length > 3 mm	1.86 (1.18–2.91)	0.007	1.82 (1.14–2.94)	0.013
Charlson Comorbidity Index	1.10 (0.89–1.36)	0.378	1.13 (0.91–1.42)	0.269

**Fig. 1:**
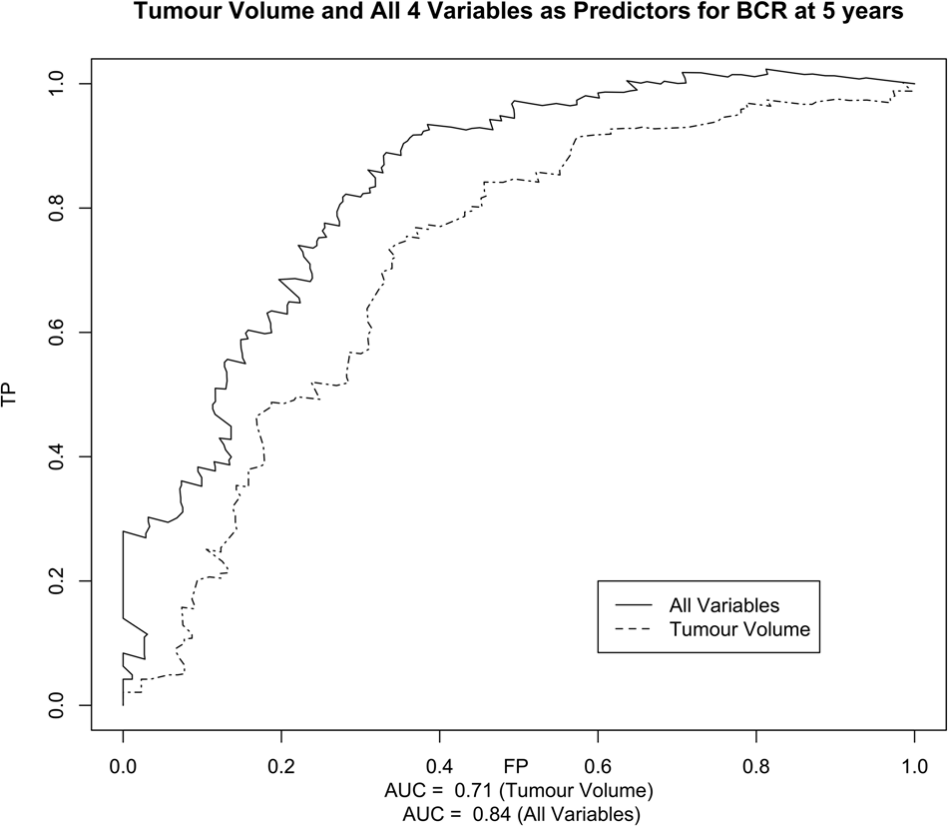
ROC curves for tumour volume and combined variables predicting BCR. ROC curves for tumour volume and all 4 signficant variables predicting BCR at 5 years in patients with high-risk prostate cancer.

ROC curves were plotted for TV alone and the four significant predictor variables using censored outcome data for BCR and TTF. When modelled over time, area under the curve (AUC) values for the prediction of both BCR and TTF peaked at 5 years. TV alone as a predictor for BCR at 5 years was 0.71 (Fig. 1). Including all significant variables as covariates increased the AUC to 0.84. The cut-off value (point of maximum TP and 1-FP) was 2.50 cm.

**Fig. 2:**
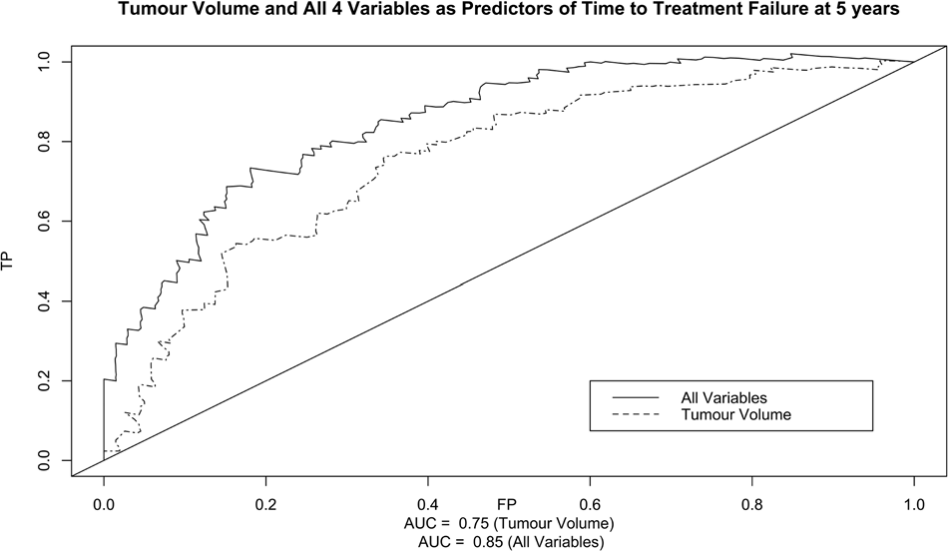
ROC curves for tumour volume and combined variables predicting TTF. ROC curves for tumour volume and all 4 signficant variables predicting TTF at 5 years in patients with high-risk prostate cancer.

For TTF AUC for TV volume alone at 5 years was 0.75 and 0.85 when all variables were included in the model (Fig. 2). TV alone resulted in a cut off value of 2.54 cm.

Despite a reduction in data points, modelling of outcomes at 10 years mirrored these results albeit with reductions in the AUC. AUC for TV alone and all variables was as a predictor for BCR 0.64 and 0.80, respectively (Supplementary Figs. 1 & 2). Likewise, AUC for prediction of TTF at 10 years was 0.72 and 0.83, respectively (Supplementary Figs. 3 & 4).

A further comparison of the model with and without TV was undertaken. A model containing using the three variables pGG, pT and margins >3 mm status had a AUC of 0.838 in comparison to the full model containing all 4 variables with an AUC of 0.843.

**Fig. 3:**
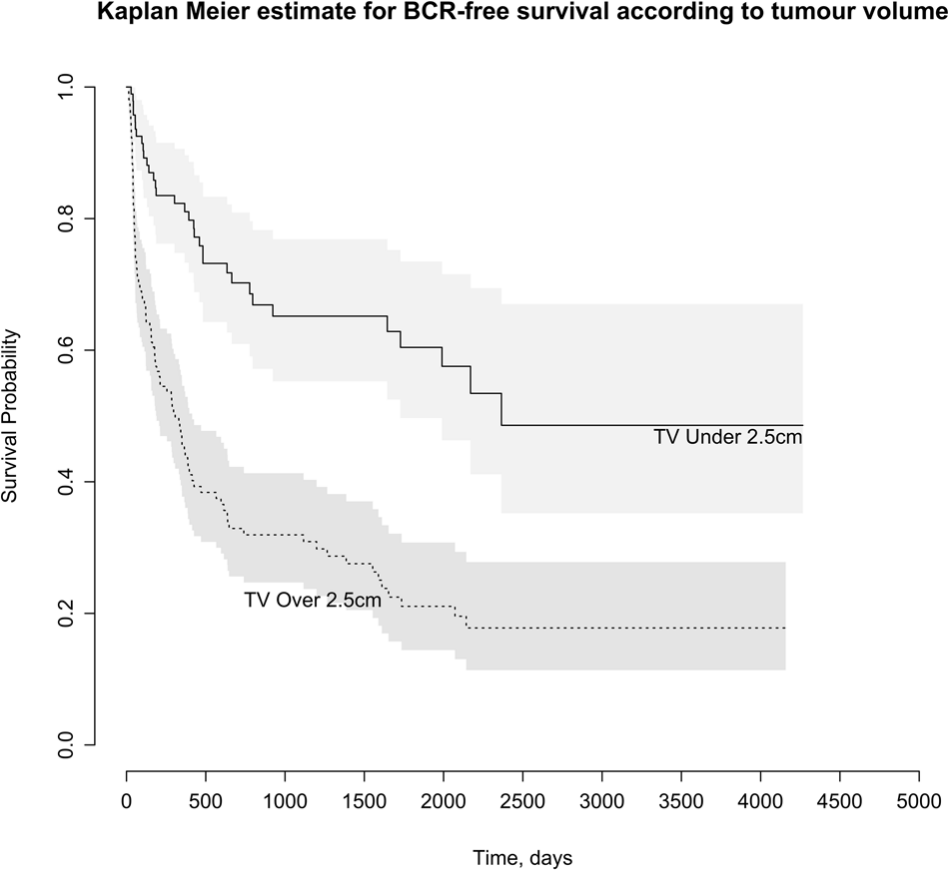
Kaplan–Meier curves for BCR-free survival. Kaplan–Meier curves for BCR-free survival stratified by tumour volume.

Kaplan–Meier estimates were plotted using a 2.50 cm TV cut off for BCR (Fig. 3). Log rank test demonstrated survival distributions were significantly different for BCR, *p* < 0.001.

## Discussion

This study demonstrates that TV measured in histopathological specimens is significantly associated with oncological outcomes following radical prostatectomy for high risk prostate cancer. However, its prognostic value when combined with known predictive variables such as pGG, pT stage and margin status is limited.

Whilst risk stratification tools for identifying low, intermediate and high-risk disease are common, exact definitions for high-risk disease are debated and outcomes may vary considerably. In certain patients, radical prostatectomy can effectively treat high-risk disease with high metastasis-free and cancer specific survival rates [12]. Various attempts have been made to identify factors that may predict outcomes in high-risk localised prostate cancer. Historically criteria set by D’Amico have been widely used although it is increasingly recognised that especially for high-risk patients, the categories may be too broad. Further systems have been promoted such as the NCCN classification used in this study. The aim of all such systems is accurately predict a specific patient’s treatment specific prognosis. In order to do so, they mostly incorporate all available prognostic information.

Various studies have refined the predictions offered by these standard models. Further subdivision of high-risk patients using the existing parameters of T stage, PSA and GG has been shown to be predict BCR and survival [13, 14]. Other studies have recommended the use of other factors such as PSAD or percentage of positive cores [15, 16]. These approaches come with a number of limitations. Accurate diagnosis in prostate cancer remains a considerable challenge despite advances in imaging modalities and biopsy technique. Previous studies have shown that even in high-risk prostate cancer, grade group may change in almost 50% of patients on final histology [17]. Results from our study reflect this uncertainty. With long-term follow up data, no preoperative factors accurately predicted either BCR or failure free survival on regression analysis. This discrepancy is likely due to the select high-risk cohort of patients.

The role of TV as a prognostic marker in prostate cancer has been assessed previously. To date TV has primarily been considered in the context of differentiating low risk significant and insignificant cancer in the preoperative setting. Correlation between post-operative TV and both other tumour parameters and oncological outcomes have been shown. Studies have demonstrated associations with PSA, Gleason grade and capsular penetration [18–20]. Likewise, post-operative TV has been shown to independently predict BCR [21–25]. Interestingly Merrill et al. found no associations for low grade tumours. These results support our finding that tumour size may predict prognosis alongside margin status, pT and pGG. Hong et al similarly found TV to be accurate predictor of PSA recurrence in high-risk prostate cancer albeit using arbitrary cut off values [26]. The interactions between TV and other pathological factors needs further exploration: is TV a surrogate marker for tumour stage and/or grade [27]? Overlapping predictive outcomes for TV and T stage in this study together with the limited additional prognostic value supports this hypothesis. Previous debate over the role of TV has been driven by a number of studies showing no correlation with outcomes [28–31]. The current study does support a possible role for TV although further work on its application is required.

Limitations to the current study need to be considered. The potential bias intrinsic to single centre retrospective analyses needs to be acknowledged. Inconsistencies in TV measure may also be relevant. Unsurprisingly computed planimetry has been shown to yield better associations than visual estimation methods [26]. Yet both total TV and volume of the primary lesion alone have been shown to be directly correlated [7]. Finally, our cohort of patients underwent heterogenous investigation protocols reflective of the developments in prostate cancer diagnostics. Potentially these may have contaminated the analysis of the preoperative investigations. Follow up to 10 years was also reduced however analysis shows evidence that the models offer consistent results in the very long term.

This study aimed to evaluate the role of TV for risk stratification in high-risk prostate cancer. Survival analysis has shown that TV has a prognostic value, but does not offer additional information in addition to pGG and pT stage. Of note TV was measured from the histopathalogical specimen; ongoing advances in imaging offer the possibility of accurately measuring TV preoperatively. TV measured on preoperative mpMRI has been shown to correlate with postoperative histological specimens although current techniques are limited by tendency to underestimate size. Even TRUS has been shown to predict TV and soon we believe that advances in prostate MRI incorporating radiomics analysis and machine learning methods offer the potential for accurate preoperative TV segmentation [2, 32, 33]. Consequently, TV may emerge as an accurate pre-operative marker for prognostication and risk stratification alongside prostate biopsy and other current tools.

## Conclusions

We conclude that reasonable experimental evidence of measured pathological TV as independent oncological prognosticator exists in this cohort of patients despite the limited value over existing markers. Our results support the following working hypothesis for future studies: segmented TV from preoperative mpMRI scans correlates with pathological TV and independently predicts oncological outcome.

## Supplementary information

Supplementary Figure Legends

Supplementary Figure 1

Supplementary Figure 2

Supplementary Figure 3

Supplementary Figure 4
