# Supplementary material for: Is tumour volume an independent predictor of outcome after radical prostatectomy for high-risk prostate cancer?
Source: Prostate Cancer Prostatic Dis. 2021 Nov 29;26(2):282–6. doi: 10.1038/s41391-021-00468-4 (PMC10247356; doi:10.1038/s41391-021-00468-4)
Supplement: Supplementary file 1 — Supplementary Figure Legends [file 41391_2021_468_MOESM1_ESM.docx]

**Supplementary Figure Legends**

Supplementary Figure 1: ROC curve for tumour volume predicting BCR at 2,4,6 and 10 years in patients with high-risk prostate cancer

Supplementary Figure 2: ROC curve for all 4 signficant variables predicting BCR at 2,4,6 and 10 years in patients with high-risk prostate cancer

Supplementary Figure 3: ROC curve for tumour volume predicting TTF at 2,4,6 and 10 years in patients with high-risk prostate cancer

Supplementary Figure 4: ROC curve for all 4 signficant variables predicting TTF at 2,4,6 and 10 years in patients with high-risk prostate cancer
