# Supplementary figures and images for: Is tumour volume an independent predictor of outcome after radical prostatectomy for high-risk prostate cancer?
Source: Prostate Cancer Prostatic Dis. 2021 Nov 29;26(2):282–6. doi: 10.1038/s41391-021-00468-4 (PMC10247356; doi:10.1038/s41391-021-00468-4)

TP

730.5

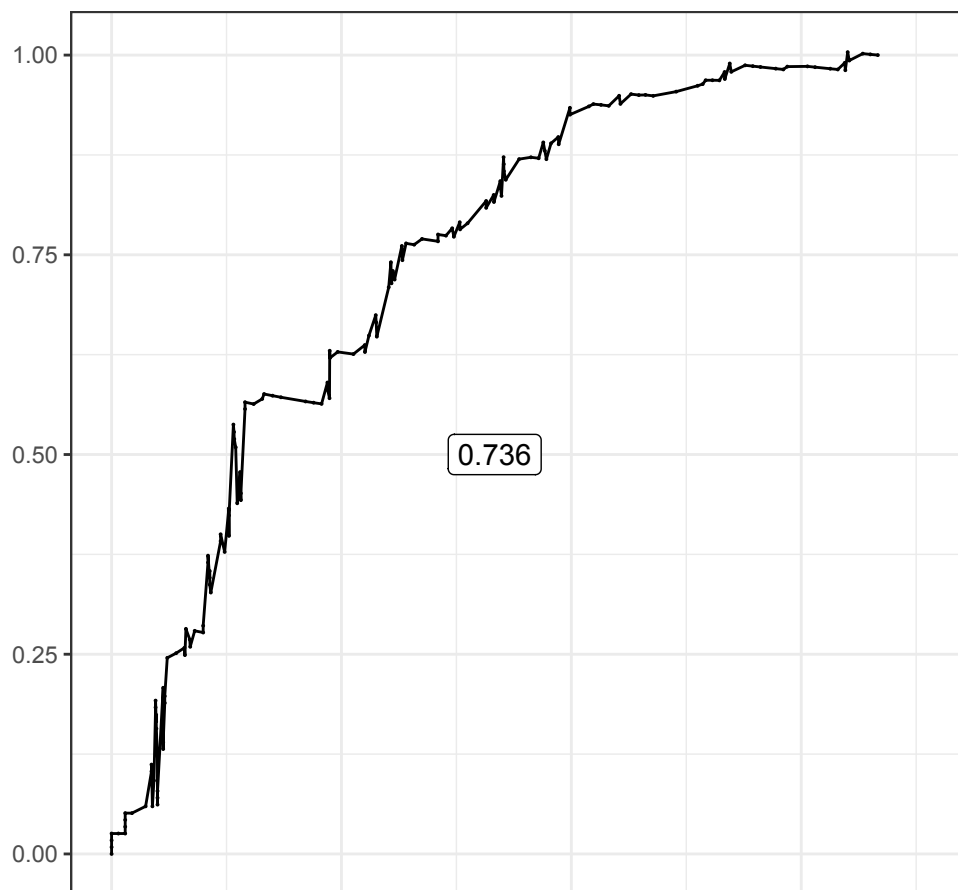

1461

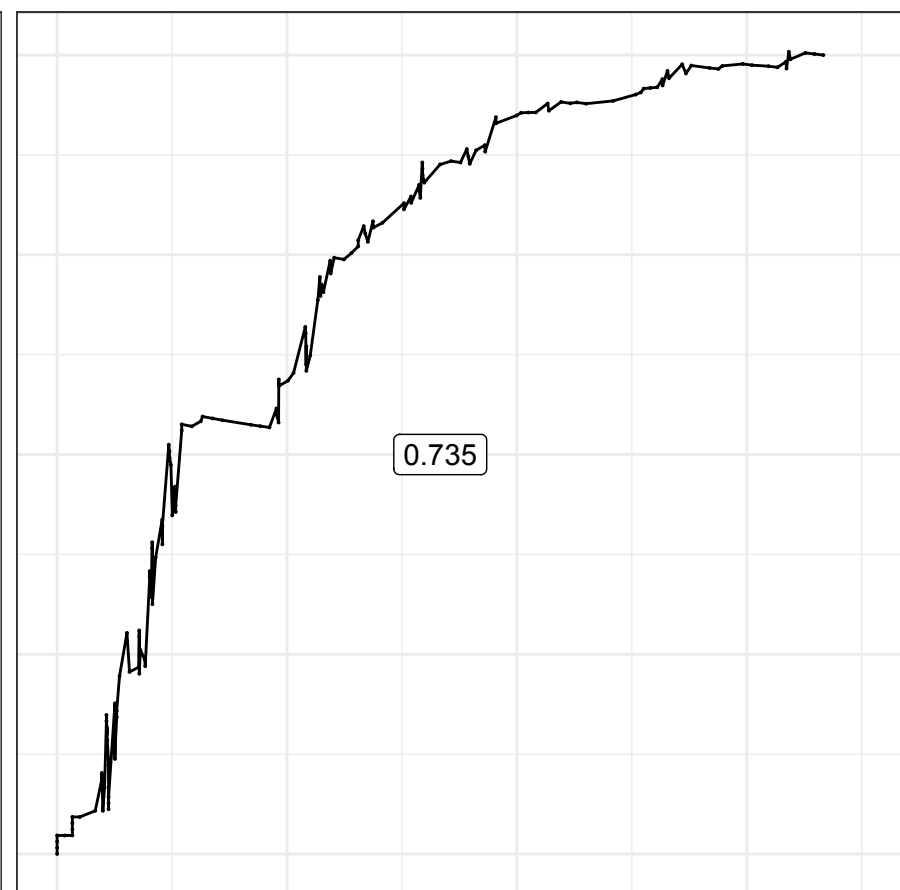

2191.5

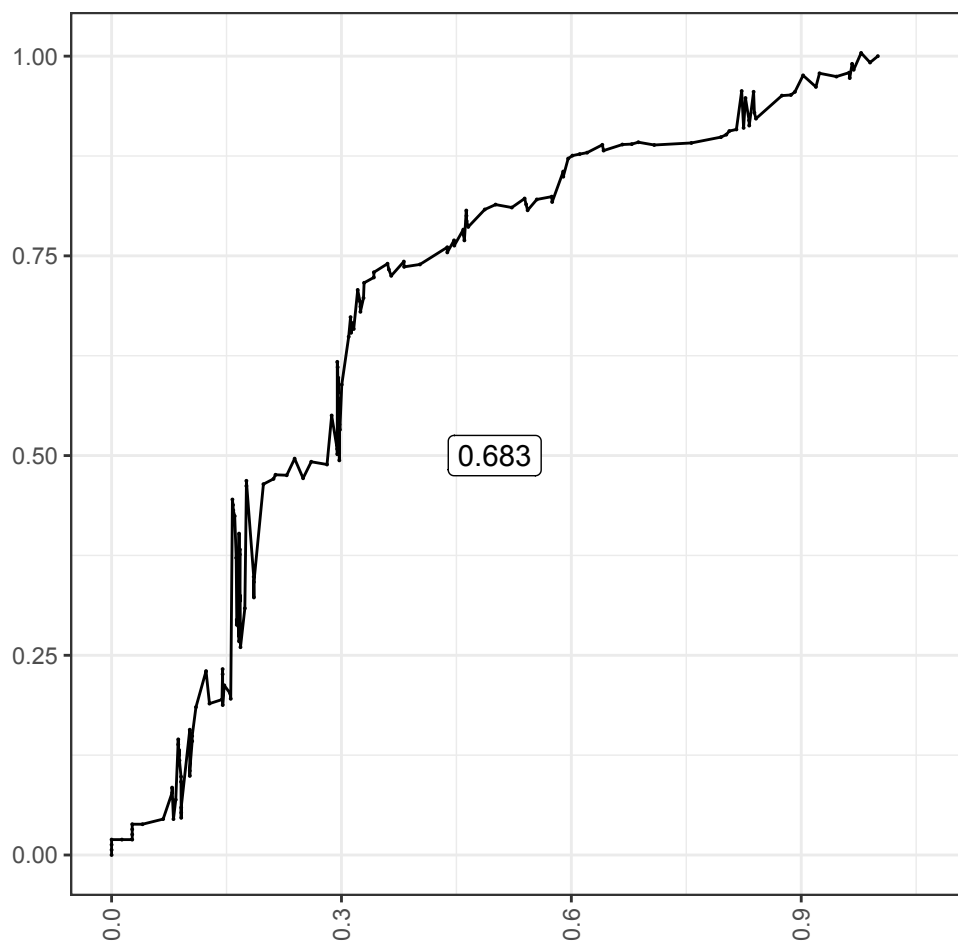

3652.5

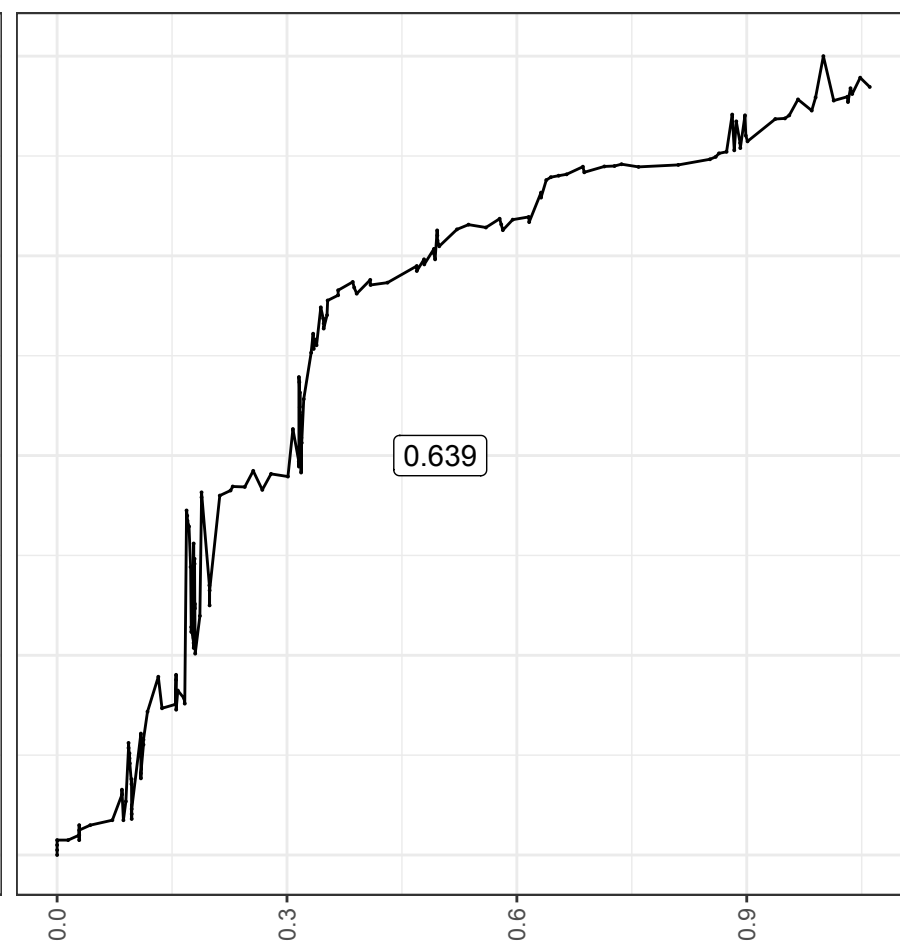

FP

Supplement: Supplementary file 2 — Supplementary Figure 1 [file 41391_2021_468_MOESM2_ESM.pdf]

TP

730.5

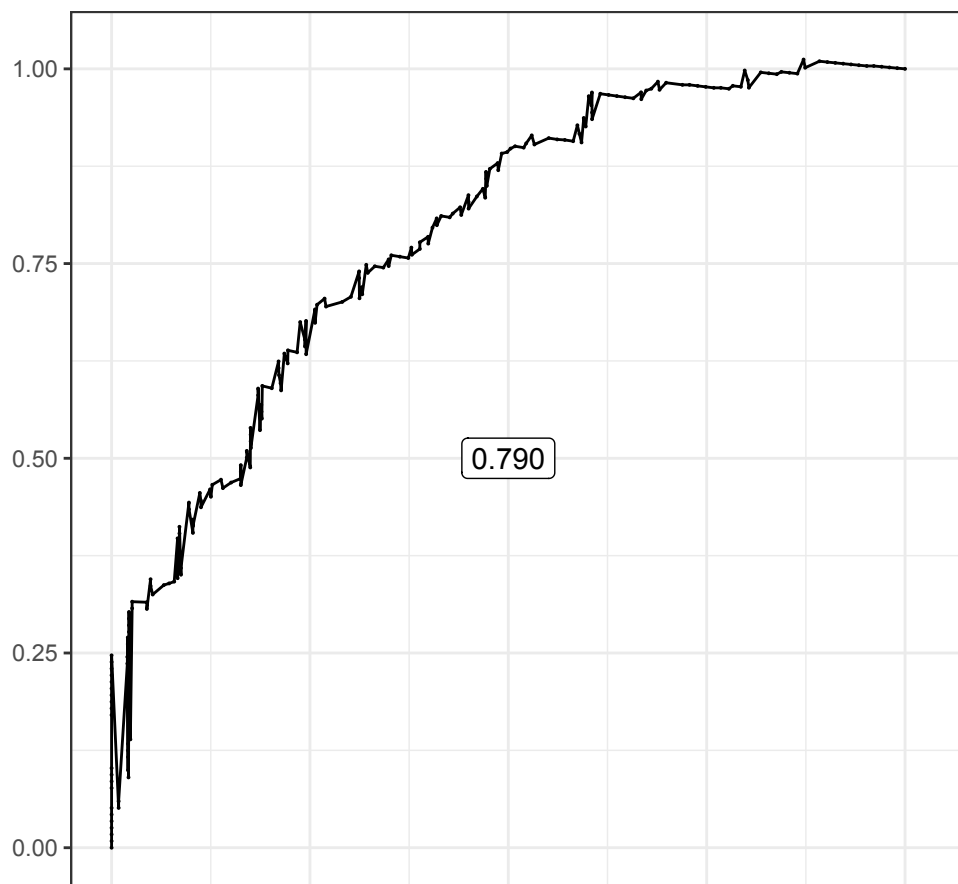

1461

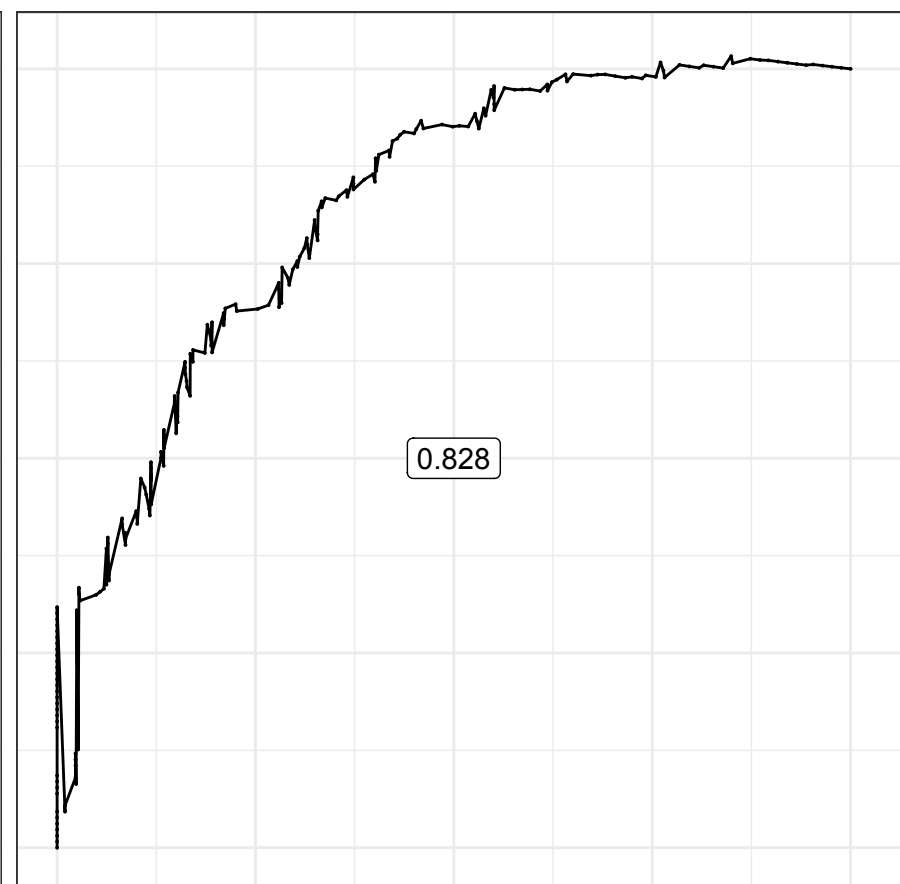

2191.5

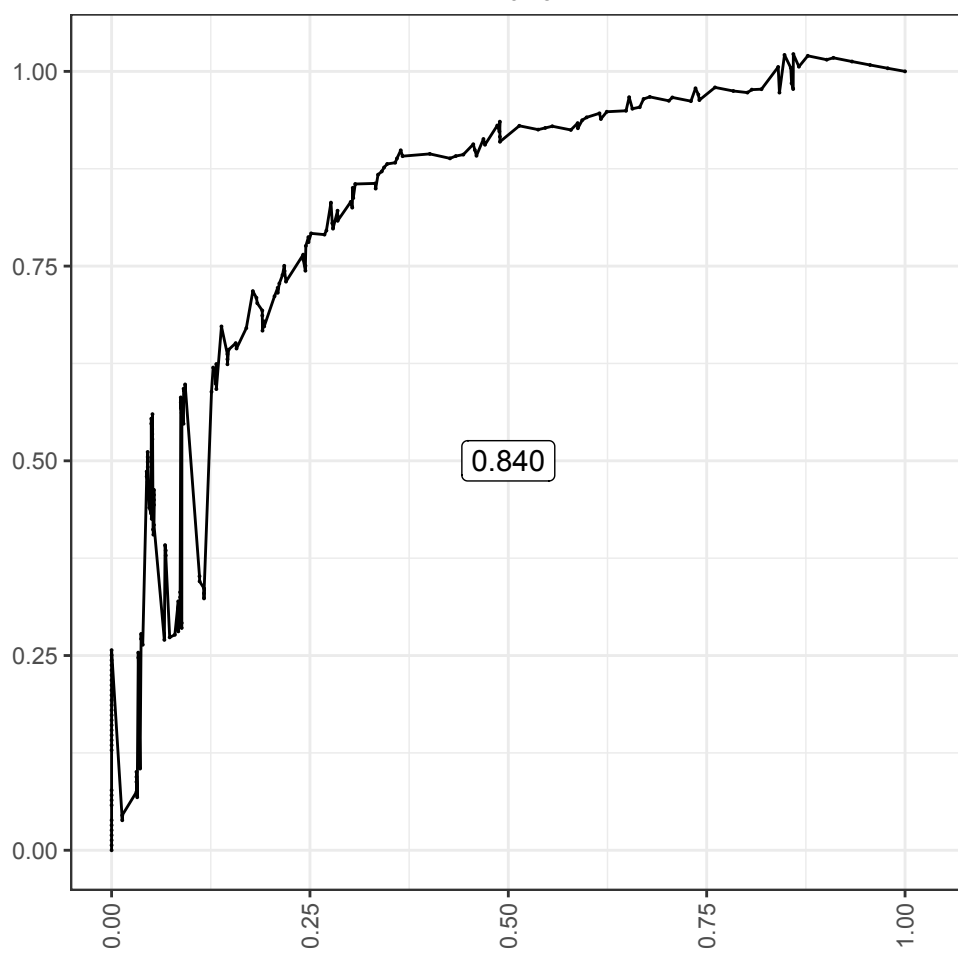

3652.5

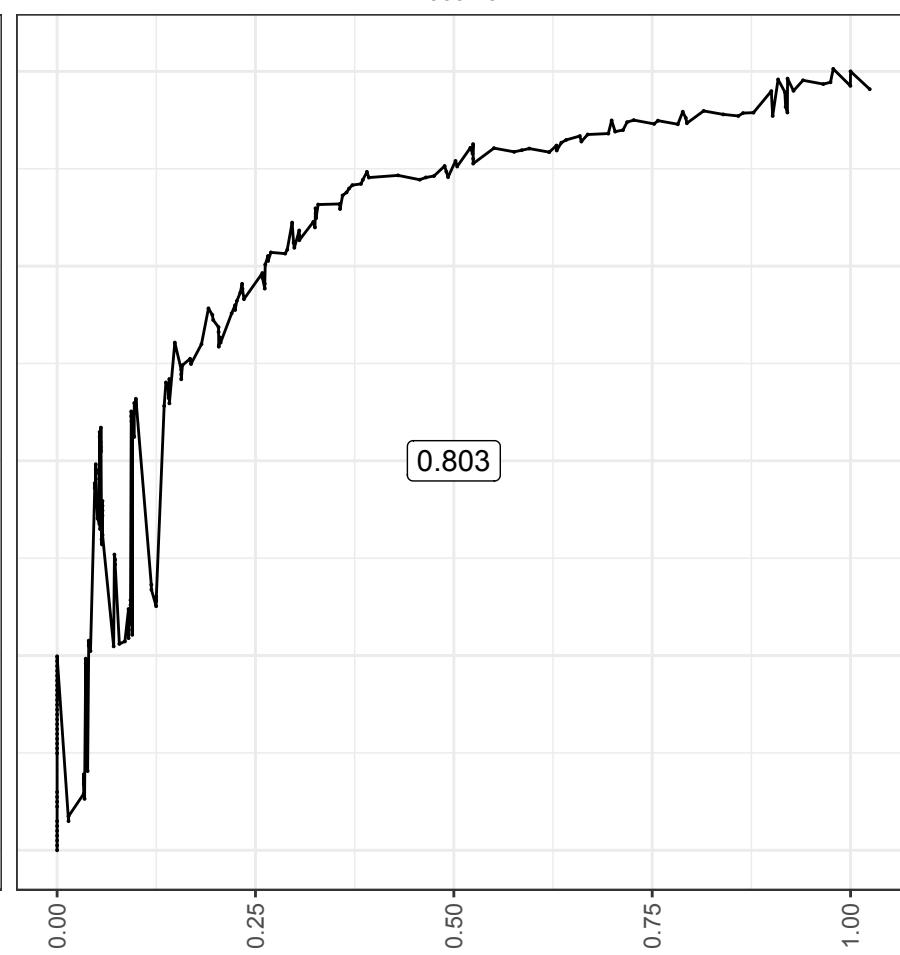

FP

Supplement: Supplementary file 3 — Supplementary Figure 2 [file 41391_2021_468_MOESM3_ESM.pdf]

TP

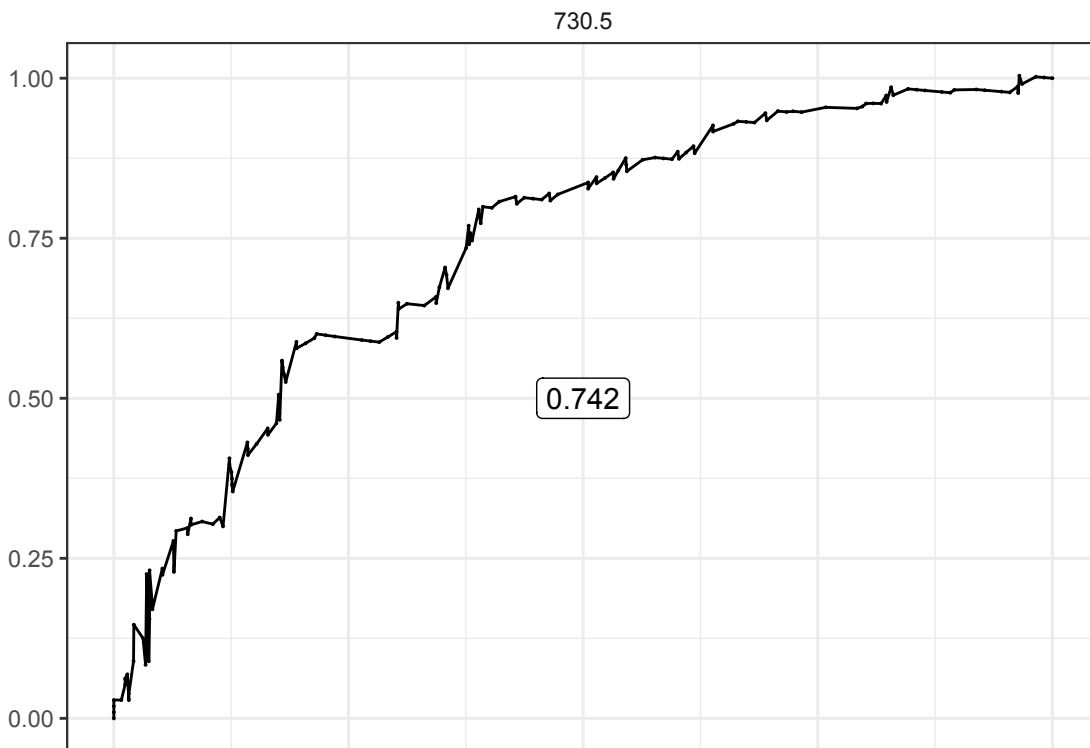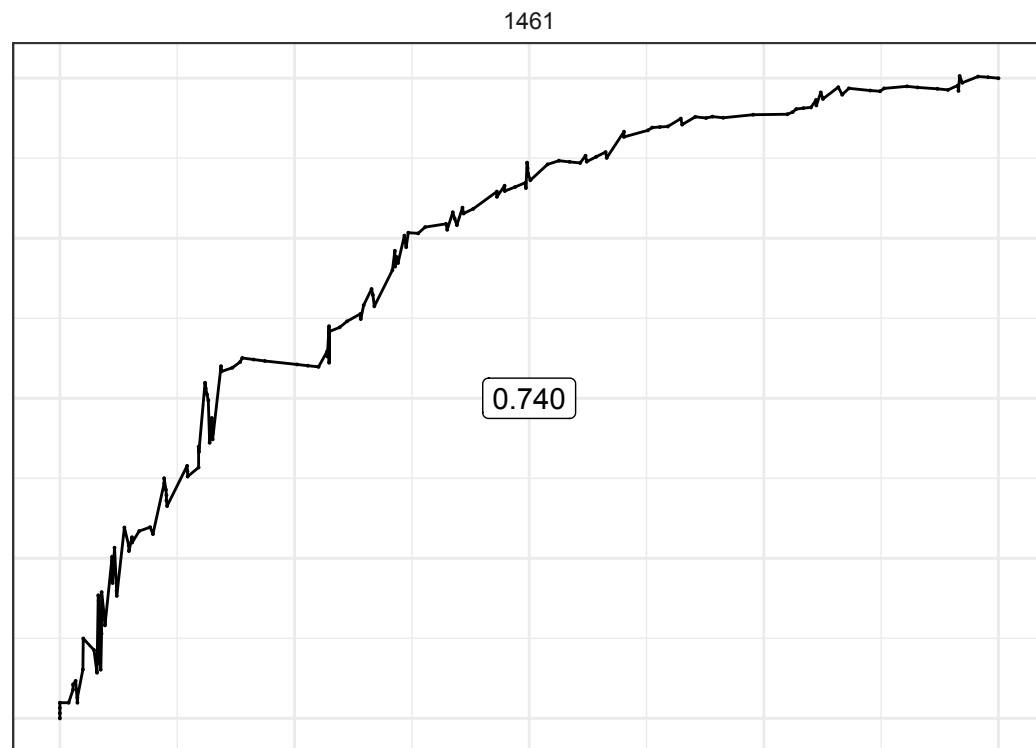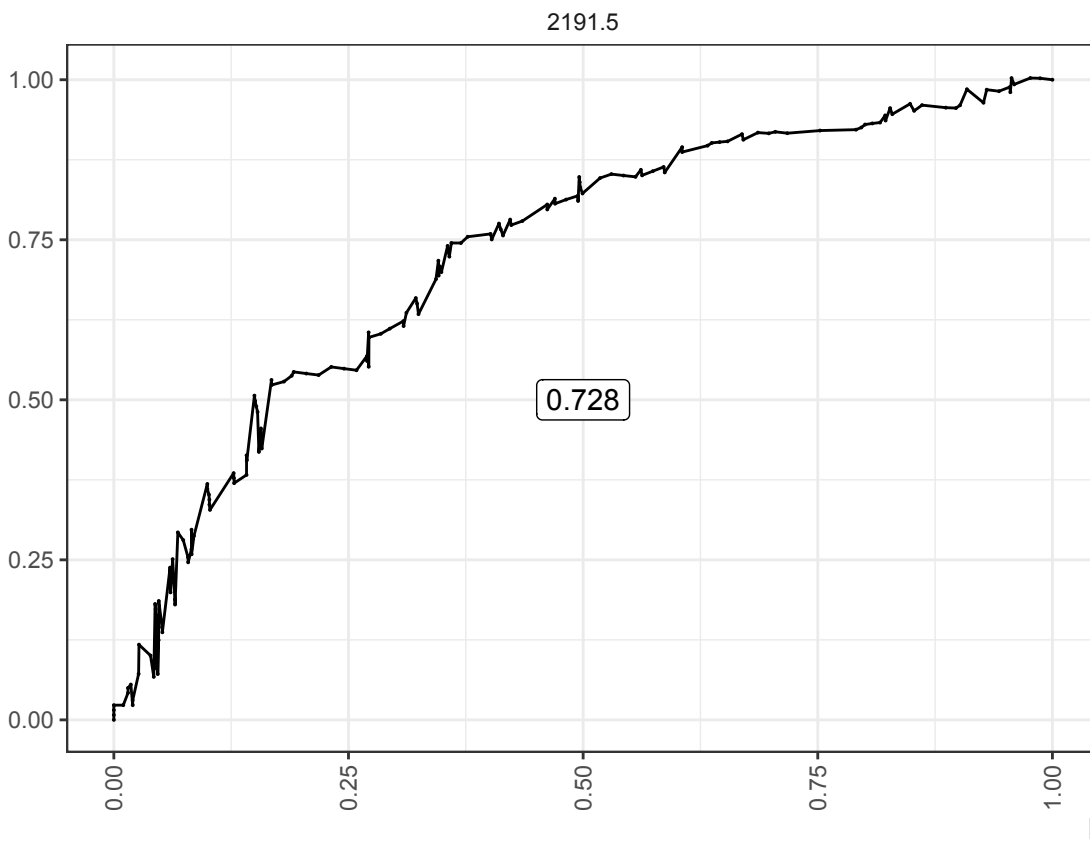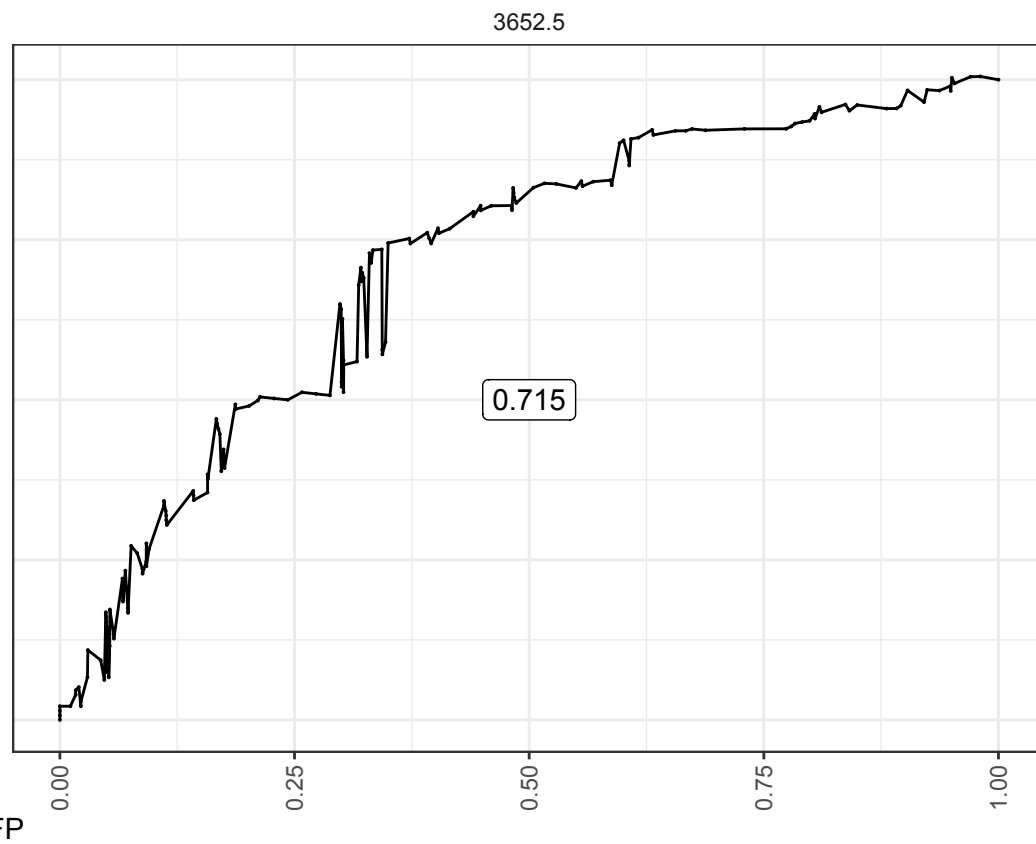

FP

Supplement: Supplementary file 4 — Supplementary Figure 3 [file 41391_2021_468_MOESM4_ESM.pdf]

TP

730.5

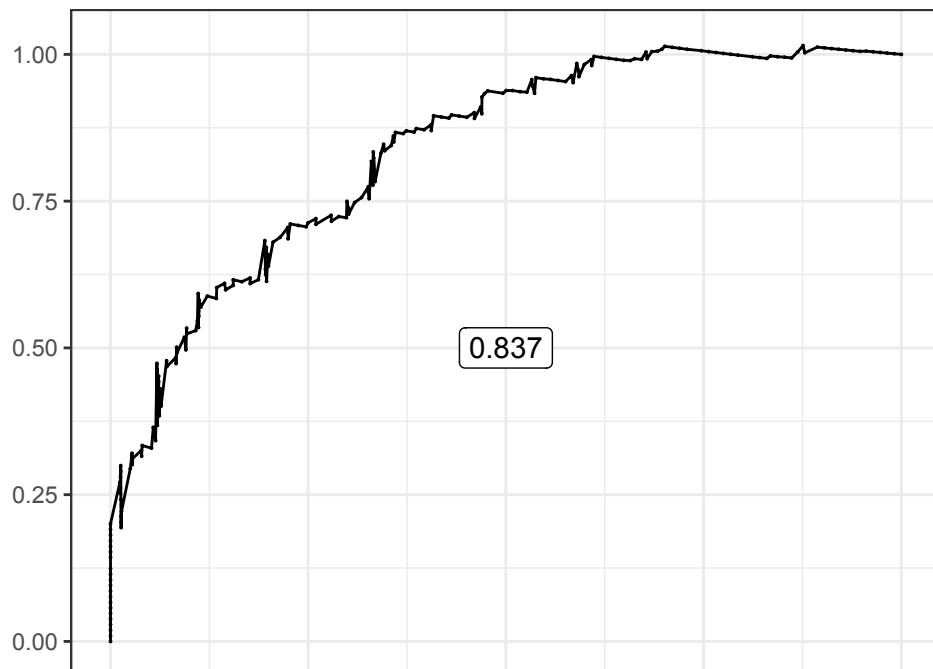

1461

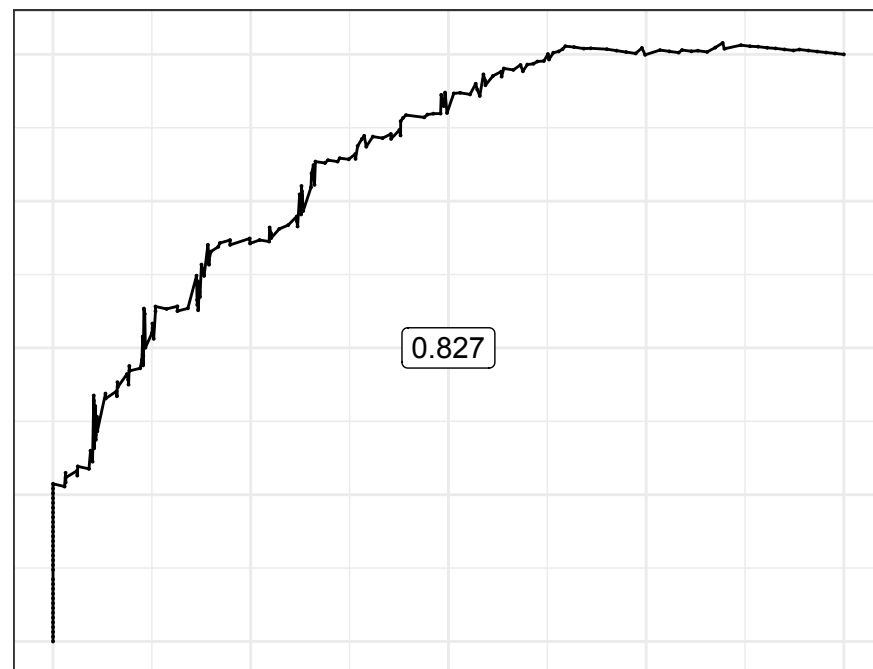

2191.5

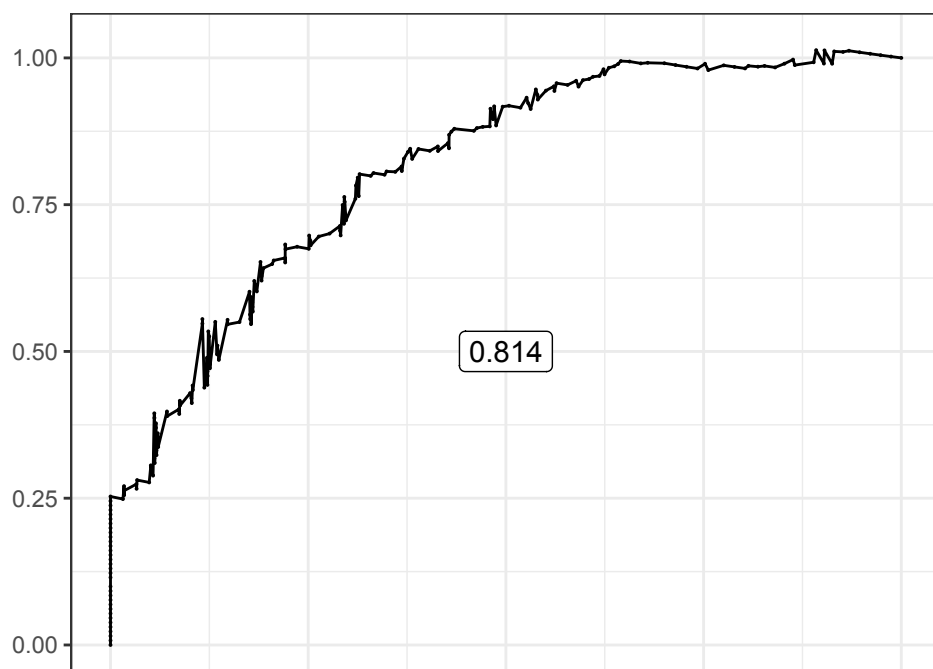

3652.5

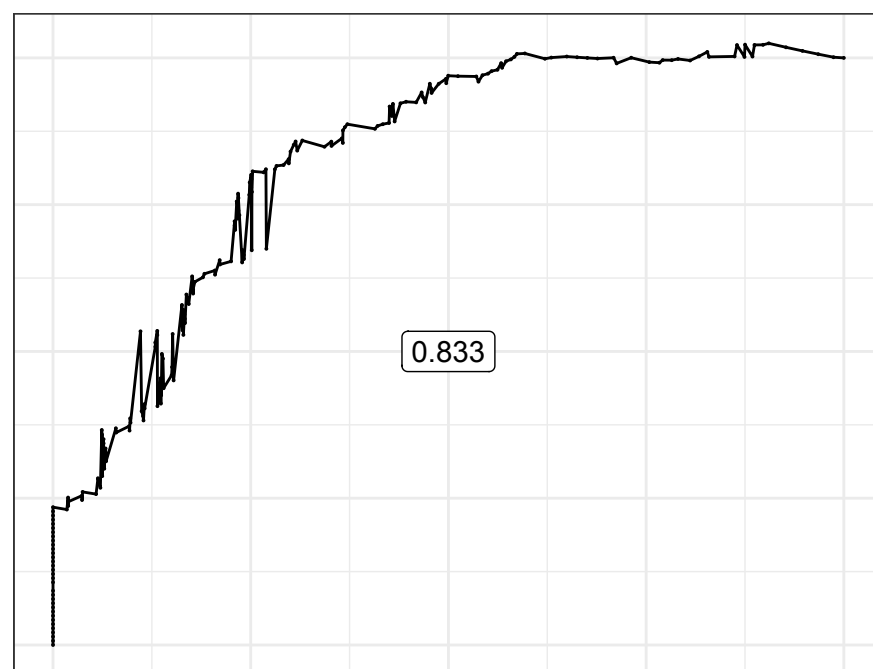

FP

Supplement: Supplementary file 5 — Supplementary Figure 4 [file 41391_2021_468_MOESM5_ESM.pdf]
